# Heterogeneous Subgreenschist Deformation in an Exhumed Sediment‐Poor Mélange

**DOI:** 10.1029/2022JB024353

**Published:** 2022-08-20

**Authors:** H. Leah, Å. Fagereng, N. Groome, D. Buchs, A. Eijsink, A. Niemeijer

**Affiliations:** ^1^ School of Earth and Environmental Sciences Cardiff University Cardiff UK; ^2^ MARUM Center for Marine Environmental Sciences University of Bremen Bremen Germany; ^3^ Department of Energy and Mineral Engineering and EMS Energy Institute The Pennsylvania State University University Park PA USA; ^4^ Department of Earth Sciences Utrecht University HPT Laboratory Utrecht The Netherlands

**Keywords:** subduction, shear zone, rheology, microstructure, Gwna, mechanics

## Abstract

Many described subduction complexes (or mélanges) exhumed from seismogenic depths comprise thick, turbidite‐dominated sequences with deformed zones containing clasts or boudins of more competent sandstone and/or basalt. In contrast, many active subduction zones have a relatively small thickness of sedimentary inputs (<2 km), turbidite sequences are commonly accreted rather than subducted, and the role of pelagic sediments and basalt (lavas and hyaloclastites) in the deforming zone near the plate interface at <20 km depth is poorly understood. Field investigation of Neoproterozoic oceanic sequences accreted in the Gwna Complex, Anglesey, UK, reveals repeated lenticular slices of variably sampled ocean plate stratigraphy (OPS) bounded by thin mélange‐bearing shear zones. Mélange matrix material is derived from adjacent OPS lithologies and is either dominantly illitic, likely derived from altered siliciclastic sediment, or chloritic, likely derived from altered volcanics. In the illitic mélange, mutually cross‐cutting phyllosilicate foliation and variably deformed chlorite‐quartz‐calcite veins suggest ductile creep was cyclically punctuated by transient, localized fluid pulses. Chlorite thermometry indicates the veins formed at 260 ± 10°C. In the chloritic mélange, recrystallized through‐going calcite veins are deformed to shear strains of 4–5 within a foliated chlorite matrix, suggesting calcite veins in subducting volcanics may localize deformation in the seismogenic zone. Shear stress‐strain rate curves constructed using existing empirical relationships in a simplified shear zone geometry predict that slip velocities varied depending on pore fluid pressure; models predict slow slip velocities preferentially by frictional sliding in chlorite, at pore fluid pressures greater than hydrostatic but less than lithostatic.

## Key Points

- Thin mélange‐bearing shear zones contain volcanic and siliciclastic material and bound lenticular slices of ocean plate stratigraphy
- Mutually cross‐cutting veins and phyllosilicate foliations suggest creep was cyclically punctuated by localized fluid‐driven brittle failure
- Rheological models predict slip partitioning into chlorite at slow slip velocities at elevated but sublithostatic fluid overpressures

## 1 Introduction

Many subduction complexes exhumed from seismogenic depths are described as thick, turbidite‐dominated sequences disrupted by duplexes and imbricate slices, and containing mélange shear zones defined by a foliated mudstone matrix surrounding clasts of more competent sandstone and/or basalt (e.g., the Chrystalls Beach, Kodiak, Sestola‐Vidiciatico, and Shimanto complexes; Fagereng, 2011b; Fisher & Byrne, 1987; Kimura & Mukai, 1991; Meneghini et al., 2009; Remitti et al., 2011; Rowe et al., 2011; Ujiie, 2002; Vannucchi et al., 2008; Wakabayashi, 2015). These exhumed subduction complexes preserve an integrated rock record that may include periods of accretion, erosion, and non‐accretion (e.g., Wakabayashi, 2021), during which the deforming thickness at any one time was a relatively small fraction of the total preserved thickness (Rowe et al., 2013). In contrast, most active subduction zones have a relatively small thickness of sedimentary inputs (36/44 segments have <2 km sediment; Heuret et al., 2012), and the degree to which pelagic sediments and basalt are incorporated into the deforming zone near the plate interface is poorly understood. These materials and their rheology may, however, be critically important; input sequences containing volcaniclastic and calcareous sediments have been sampled by drilling seaward of the Costa Rica and Hikurangi subduction zones (Harris et al., 2013; McKiernan & Saffer, 2006; Wallace et al., 2019), where it has been suggested they may become entrained within the décollement (Barnes et al., 2020) and deform in distinct lithologically controlled styles at depth (Ikari et al., 2013). Heterogeneity within the incoming sequence and the plate interface is also thought to contribute to complex slip behaviors, such as slow slip and tremor, possibly in concert with high pore fluid pressures (Agard et al., 2018; Barnes et al., 2020; Beall et al., 2019; Boulton et al., 2019; Condit & French, 2022; Fagereng, 2011b; French & Condit, 2019; Tulley et al., 2022).

A further contributor to this heterogeneity may be seamounts (and other geometrical irregularities), which are present seaward of many subduction zones and have also been associated with complex slip styles (Bell et al., 2010; Bonnet et al., 2019; Shaddox & Schwartz, 2019; Sun et al., 2020; Todd et al., 2018; Wang & Bilek, 2011). Seamounts can be accreted and exposed at margins with less siliciclastic/volcaniclastic turbidites and/or thinner incoming sediment sequences (Buchs et al., 2011), where deformation of basalts and their sedimentary cover is relatively more important. Although difficult to assess with seismological techniques in active, modern margins, insights on how heterogeneous rock types influence deformation style and slip partitioning on the plate interface of subduction zone seismogenic zones may be derived from the heterogeneous composition and map‐ to micro‐scale structure of ancient plate interface shear zones from the rock record. Here we present data from the Gwna Complex at Llanddwyn Island, Anglesey, UK, a Neoproterozoic subgreenschist subduction complex containing narrow imbricated shear zones with <100 m thick sedimentary rock packages between larger lenses of pillow basalts with subordinate hyaloclastites. We characterize the role of heterogeneous volcanic and pelagic sedimentary material in plate interface deformation, and show that altered volcaniclastic materials can be of similar strength to under‐thrust siliciclastic sediments when subject to plate interface deformation at temperatures <300°C. We use a rheological model (following French & Condit, 2019) based on published rock deformation experiments and our geological observations to derive relations between stress, strain rate, and fluid pressure for various shear zone components. Key conclusions include that increasing fluid pressure allows increasing slip rate by facilitating a change in the material that accommodates slip (from illite‐quartz to chlorite) and the dominant deformation mechanism (from frictional‐viscous creep to frictional sliding). Because the subduction thrust is a multi‐component shear zone of finite thickness throughout the seismogenic zone (Fagereng & Beall, 2021; Rowe et al., 2013), and incoming lithological layering can be traced to control slip style (Barnes et al., 2020), we suggest these conclusions can be upscaled from the exhumed example of the Gwna Complex and are important at the margin scale.

## 2 Geological Setting of the Gwna Complex

The Gwna Complex (also referred to as the Gwna Mélange or Gwna Group) is an Ediacaran to Cambrian‐age collection of mixed‐grade metasediments and meta‐igneous lithologies found throughout much of Anglesey (Greenly, 1919), an island off the north‐west coast of Wales. The Gwna Complex has been interpreted as related to ancient subduction (Kawai et al., 2007; Maruyama et al., 2010; Schofield et al., 2021); it is the oldest preserved material that accreted to the upper plate during south‐eastward subduction of oceanic crust beneath Avalonia from 488 to 448 Ma (Kawai et al., 2007). The preserved sequence is overall downward‐younging, based on formation ages of subduction‐related volcanic layers, inferred to reflect tectonic underplating (Kawai et al., 2007). The Gwna Complex was subject to low‐grade (zeolite or prehnite‐pumpellyite facies) metamorphic conditions (Kawai et al., 2007), with the exception of a blueschist unit found across central southern Anglesey (Gibbons & Horák, 1990). Peak burial pressures range from approximately 0.3 to 1 GPa for this range of metamorphic conditions (Kawai et al., 2006). The distinct low‐grade and blueschist units are thought to have been emplaced along high‐angle NE‐SW trending strike‐slip faults with sinistral senses of shear (Gibbons & Horák, 1990), that may have accommodated significant lateral translation during subduction (Schofield et al., 2021).

**Figure 1:**
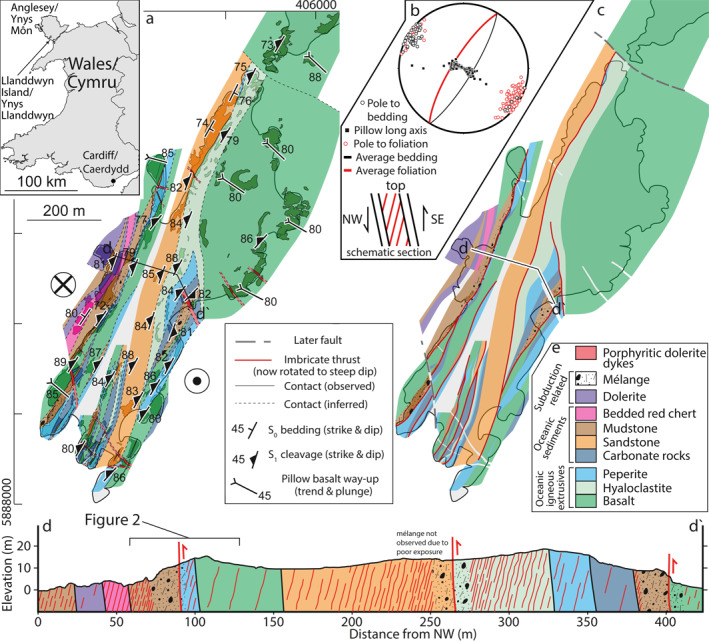
Map and section of Gwna Complex lithologies and structure at Llanddwyn Island, Anglesey, Wales, UK. Parts show (a) lithology distribution throughout the complex, inset shows location of Llanddwyn Island in north Wales, (b) equal area, lower hemisphere stereonet showing bedding and foliation measurements from throughout the area covered in (a), (c) stratigraphic units and locations of imbricate thrusts, (d) cross‐section across Llanddwyn island showing thrust positions and large‐scale lithological texture (highlighted area shows position of Figure 2), (e) approximate ocean plate stratigraphy reconstructed from the area, variably sampled in lenses between imbricate thrusts. Grid coordinates in (a) are in UTM zone 30U.

Recently, a reinterpretation of lithologies across Anglesey has resulted in the Gwna Complex at Llanddwyn Island being reclassified as the Llanddwyn Island Volcanic Member, part of a mega‐conglomerate called the Bodorgan Formation (Schofield et al., 2021). The Bodorgan Formation is thought to record NW‐vergent folding and thrusting associated with Early Devonian subduction, and the Llanddwyn Island Volcanic Member is said to lie in strands of the strike‐slip Berw Fault Zone which extends across Anglesey (Schofield et al., 2021). While recognizing this broader context, our current work focuses on processes of subduction deformation recorded in structures with a dip‐slip shear sense clearly preserved at Llanddwyn Island (Figure 1; Kawai et al., 2008; Maruyama et al., 2010).

The Gwna Group at Llanddwyn Island is lithologically consistent with ocean plate stratigraphy (OPS), defined by (Isozaki et al., 1990) as sediments deposited upon oceanic crust in an open‐ocean environment, in our case including lavas and volcaniclastics, cherts, limestones, mudstones, and coarser, foliated siliciclastic sedimentary rocks (Greenly, 1919; Maruyama et al., 2010). Pillow lavas at Llanddwyn Island have both MORB and OIB chemistries (Saito et al., 2014; Thorpe, 1993) and are thought to be the basal unit of repeated OPS imbricated in a duplex configuration between floor and roof thrusts (Maruyama et al., 2010). Massive carbonates are found throughout the island, commonly adjacent to altered basalts (Maruyama et al., 2010). Peak metamorphic conditions were subgreenschist facies, typically meaning temperatures less than 300°C at burial depth less than 20 km (Kawai et al., 2006). Constraints on the depositional and deformational ages of these lithologies are uncertain; Neoproterozoic carbonates from the Gwna Complex in the north of Anglesey likely formed 880‒750 Ma (Horák & Evans, 2011) and Cambrian siliciclastic sediments on Llanddwyn Island were deposited after a maximum U‐Pd depositional age of 550 ± 24 Ma (Asanuma et al., 2017). Subduction of the Gwna Group likely began before 600 Ma, accreting the Gwna Complex at Llanddwyn Island after the deposition of the coarsest siliciclastic lithologies after 550 Ma (Asanuma et al., 2017) and continuing until the Ordovician (448 − 488 Ma; Kawai et al., 2007). This relatively long‐lived, inferred history of convergence relates to the larger‐scale complex Cambrian‐Ordovician subduction‐collision Appalachian‐Caledonian belt, where post‐tectonic steepening of subduction‐related structures could relate to collisions of arcs and microcontinents (c.f. van Staal et al., 1998). However, there is no evidence for post‐subduction collisional tectonics on Llanddwyn Island, such as a thermal overprint or introduction of arc or continental rock fragments, but rather two distinct structural assemblages ‐ an early reverse‐sense shear deformation distributed on several structures that duplicated ocean‐floor stratigraphy, and a later strike‐slip overprint on discrete, overprinting, steep structures. We therefore avoided outcrops where later strike‐slip faulting has overprinted the earlier, subduction‐related structures, and note that current structures have rotated into a generally steeper dip than that at which they were most likely active, a rotation that we interpret as passive block rotation either in the accretionary wedge or as part of bulk strike‐slip deformation.

Exhumation of the Gwna Complex at Llanddwyn Island is associated with an antiformal stack of variable metamorphic grade, up to blueschist facies (Kawai et al., 2007; Schofield et al., 2021). Though difficult to discern due to several later high‐angle strike‐slip faults, the sub‐blueschist facies units of the Gwna Complex at Llanddwyn Island have been interpreted to sit structurally above this blueschist unit (Kawai et al., 2007). High‐angle normal faults found throughout Anglesey are thought to have accommodated much of the exhumation‐related deformation throughout the Gwna Complex, but generally cross‐cut and juxtapose subunits of the Precambrian to Ordovician sequence on a much larger scale than is considered here. Away from these high‐angle normal faults, pre‐exhumation structures resulting from subduction‐related deformation are preserved. Ordovician‐age supra‐subduction dykes have intruded cherts on the NW side of Llanddwyn Island (Figures 1a and 1c). Finally, north‐south trending dolerite dykes found throughout Anglesey are associated with the opening of the Atlantic Ocean and intruded around 55 ± 1.7 Ma (Allott & Lomax, 1988; Hailwood et al., 1992; Kirton & Donato, 1985). Similar to strike‐slip structures on the island, these later dykes were avoided in the field, where possible.

## 3 Data Collection

Mapping and sampling was carried out across Llanddwyn Island, a UK National Nature Reserve and Site of Special Scientific Interest, also part of the GeoMôn UNESCO Global Geopark. This study focuses on shear zones found on the island and presents a simplified version of a more complete geological map and lithological interpretation that extend onland and will be presented in another contribution (Groome et al., 2022). Orientation data dominantly comprise bedding within lenticular slices and foliation measurements within thin (<20 m) zones of more intense macroscopically ductile deformation. Pillow basalt way‐up indicators were also measured, along with the length and orientation of the longest axes of a subset of pillows. Samples were collected under the guidance of Natural Resources Wales and GeoMôn Geopark to minimize impact in this Site of Special Scientific Interest. The samples were cut perpendicular to foliation or bedding and parallel to the long axes of basalt pillows measured in the study area to produce polished thin sections, typically in a subvertical plane oriented NW‐SE.

Thin sections were coated with 5–8 nm of carbon to prevent charging before backscatter electron (BSE) imaging and energy dispersive spectroscopy (EDS) mapping were carried out at the School of Earth and Environmental Sciences in Cardiff University with a Zeiss Sigma HD Field Emission Gun Analytical scanning electron microscope (SEM) fitted with two Oxford Instruments 150 mm^2^ energy dispersive X‐ray spectrometers and a Nordlys EBSD system with Oxford Instruments Aztec software. EDS mapping was carried out at 15 or 20 keV accelerating voltage, a beam current of 4.3 nA, an aperture of 120 μm, a working distance of 8.9 mm, and stepsizes of 1.2 or 5 μm. Recrystallized calcite veins were mapped with electron backscatter diffraction (EBSD) on thin sections polished with colloidal silica and tilted at 70° to the electron beam at a stepsize of 2 μm, a working distance of 13 μm, an accelerating voltage of 20 keV, a beam current of 8.5 nA, and a 120 μm aperture. Raw EBSD backscatter patterns were processed using Oxford Instruments Aztec software with a gain of 5 and 2 × 2 binning.

## 4 Observed Structure of the Gwna Complex at Llanddwyn Island

Exposures across Llanddwyn Island are dominantly coastal, with irregular 5–200 m thick lenticular slices of ocean plate stratigraphy (OPS) cropping out parallel to the NE‐SW trend of the island (Figure 1a). Bedding and foliation are subvertical with local overturned beds and strike NE‐SW; however, dip angles between the two differ on average by ∼20° to create an asymmetry consistent with SE‐side up, dip‐parallel shear (Figure 1b). Pillow basalts throughout the island have subvertical layering and indicate younging toward the SE, consistent with previous work (Maruyama et al., 2010). Hyaloclastites produced by subaqueous fragmentation of lava flows locally occur at the base of pillow lavas or are juxtaposed against other lithologies in the north and central parts of the island. Local basalt‐carbonate breccias form a minor lithological component between the pillow lavas and their carbonate sediment cover and are interpreted as peperite (i.e., syn‐volcanic breccias that formed due to the interaction of lava flows with unlithified carbonate sediments on the seafloor). Deformation is localized on <20 m thick mélange‐bearing shear zones, which anastomose throughout the study area at the margins of lenticular slices of OPS (Figures 1c and 1d). Pillow basalts adjacent to mélange‐bearing shear zones are commonly elongated with their long axis subparallel to the dip of bedding and foliation, corroborating a dip‐slip shear sense (Figure 1b). Due to their low angle to bedding we assume these shear zones were active at a lower angle and have been rotated during later exhumation. We will therefore refer to them as thrusts for the remainder of this work.

### 4.1 Composition of Imbricated Mélange Shear Zones

**Figure 2:**
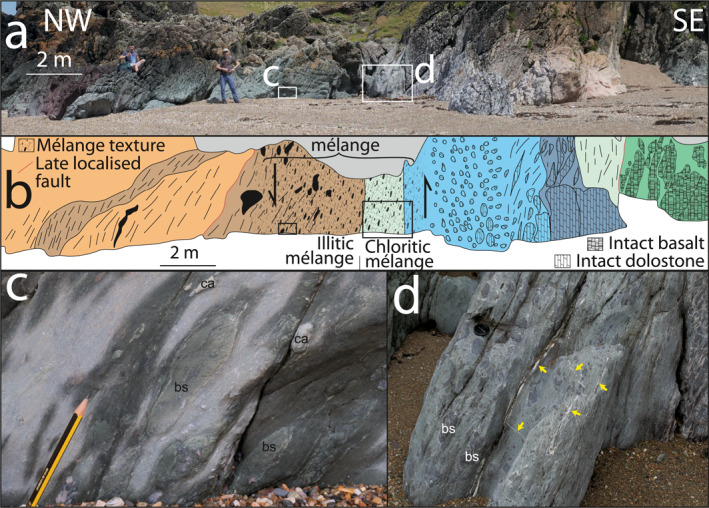
Field‐scale relation of mélange units to imbricated stratigraphy. Panels show (a) panorama of imbricate thrust exposure between two lenses of intact stratigraphy, (b) schematic sketch with indicative textures across lithologies found in (a), color scheme is the same as Figure 1. (c) close‐up of illitic mélange containing clasts of carbonate (ca) and basalt (bs), and (d) field photo of chloritic melange containing clasts of basalt (examples labeled bs) and several veins (examples at yellow arrows). Approximate location of (a) shown in Figure 1d.

The mélange shear zones on Llanddwyn Island occur as non‐planar tabular volumes of locally variable thickness (5–20 m). The SE‐up, NW‐down shear sense of the mélange‐bearing thrusts defined by bedding‐foliation asymmetry (Figure 1b) is used to define the NW side as the footwall and the SE side as the hanging wall. Mélange on the footwall side of the thrusts generally comprise sandstone clasts in a dominantly illite matrix, likely derived from sandstone and mudstone from the top of the OPS. On the hanging wall side the mélange generally comprises a chlorite matrix with clasts of altered basaltic material, likely derived from basaltic volcanics (pillow lava and hyaloclastite) at the base of the OPS (Figure 2). As such, the mélanges described here contain blocks derived from either shear zone wall, and lack exotic blocks. This repetition of OPS astride subparallel mélange shear zones of consistent shear sense defines an irregular imbricated structure (Figure 1d), similar to what has been observed in other subduction‐related mélanges (Fisher & Byrne, 1992; Kimura et al., 2012; Onishi et al., 2001; Regalla et al., 2018; Shibata et al., 2008; Wakabayashi, 2015).

**Figure 3:**
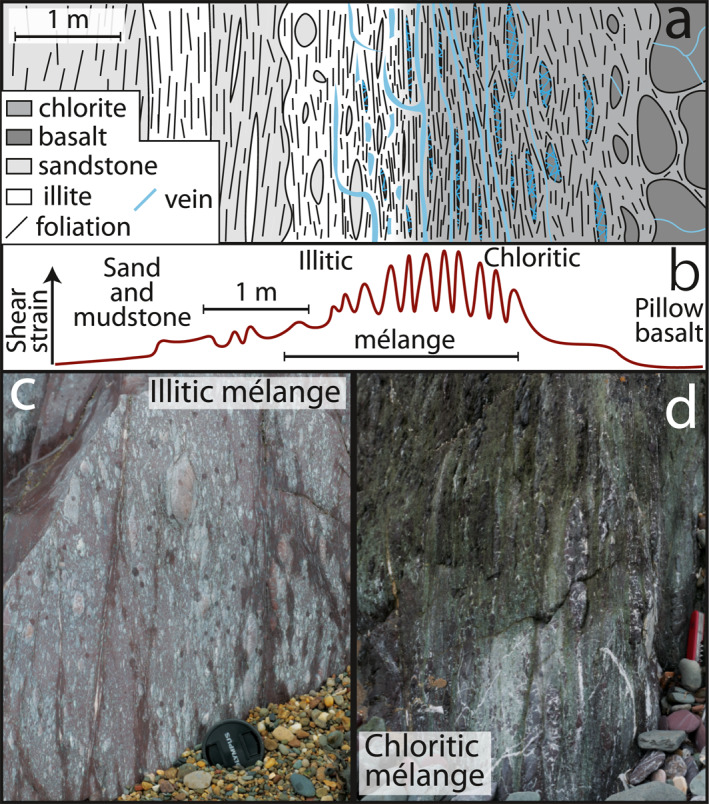
Schematic sketch of an idealized imbricated mélange shear zone and photos of its constituent units. (a) schematic sketch of lithology and texture, (b) qualitative interpreted shear strain distribution where matrix strain is estimated from foliation intensity, clast strain from clast aspect ratios, and the matrix is generally assumed to have higher strain than the clasts (*c.f.* Fagereng & Sibson, 2010), (c) field photo of illitic mélange texture (lens cap is approx. 6 cm across), (d) field photo of chloritic mélange texture (penknife is ∼9 cm long).

Most mélange shear zones have incorporated material from both the hanging wall and the footwall in block‐in‐matrix textures up to 10 m wide (Figure 2). Elongate blocks within the matrix are generally derived from more competent lithological layers (e.g., sandstone clasts from the footwall and basaltic clasts from the hanging wall; Figures 2b–2d and 3). The matrix has a foliation that wraps around blocks and is dominated by phyllosilicates; namely illite and chlorite. The thickness of footwall sediments (10 − 100 m) is commonly greater than that of hanging wall volcanics (<50 m) adjacent to mélange shear zones (Figures 1a, 1c and 1d); the change from block‐in‐matrix texture to sedimentary sequence is also more gradational (2 − 10 m) than that to relatively undeformed volcanics (<5 m; Figure 2).

## 5 Deformation Texture and Composition of Imbricated Mélange Shear Zones

**Figure 4:**
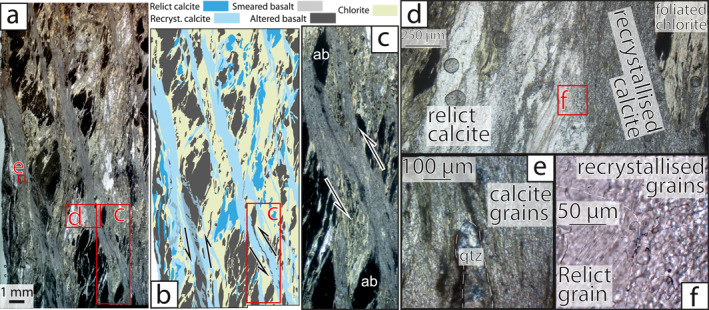
Deformation microstructures of chloritic mélange. Thin section photo mosaic (a) and associated sketch (b) show through‐going calcite veins cross‐cutting the dominantly foliated chlorite matrix which encompasses clasts of altered basalt. (c) recrystallized calcite veins have smeared out altered basalt labeled ‘ab’ to shear strains of ∼4.5. (d) away from through‐going veins, calcite is not as completely recrystallized, (e) minor quartz is present in some calcite veins, (f) relict calcite grains contain subgrains of similar size to adjacent recrystallized grains.

**Figure 5:**
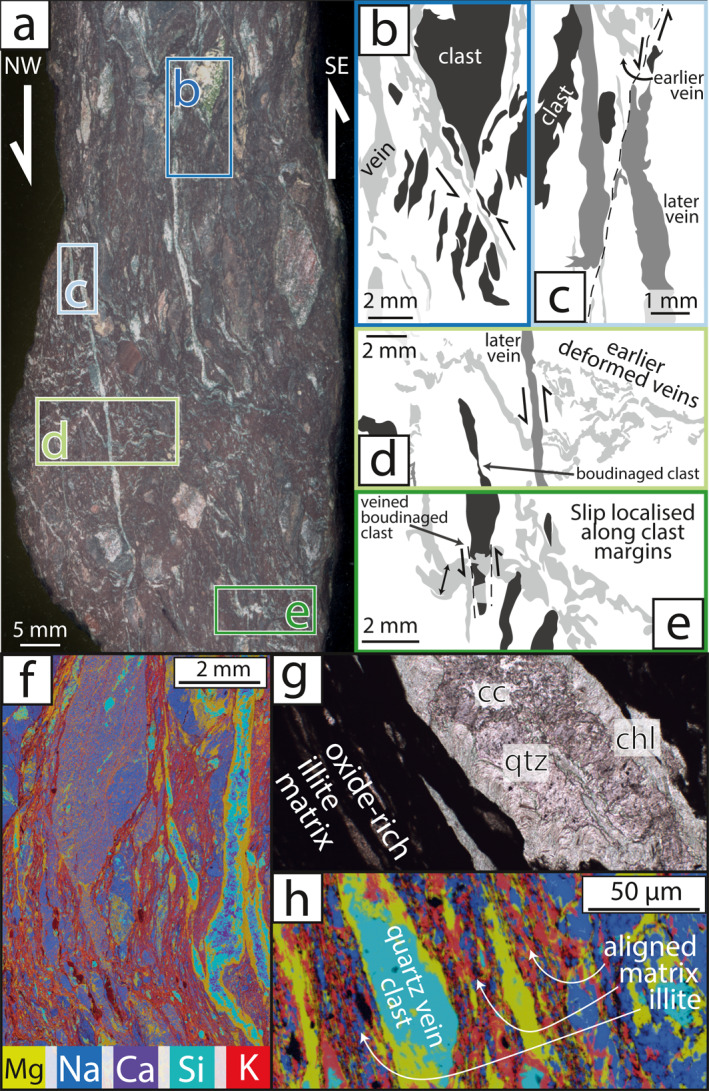
Deformation microstructures of illitic mélange. Hand specimen cut face (a) and associated sketches show (b) hybrid shear vein localisation adjacent to clasts, (c) foliation‐parallel veins cut by localized shear bands, (d) foliation‐parallel veins cutting deformed foliation‐oblique veins adjacent to boudinaged clasts, (e) shear band localisation adjacent to a boudinaged clast infilled with vein material. SEM‐EDS maps and photomicrographs show (f) hybrid shear quartz‐calcite‐chlorite veins cross‐cutting an illitic matrix, (g) hybrid shear veins bordered by greenish chlorite adjacent to the matrix, (h) clasts within the matrix derived from fragmented quartz‐calcite‐chlorite veins.

Within mélange shear zones, microstructures are dominated by clast‐in‐matrix textures (Figures 3, 4, 5). Phyllosilicates (either chlorite or illite) between clasts are fine‐grained (<20 μm), forming a sub‐planar foliation that wraps around less deformed clasts (Figures 4 and 5). There are distinct differences between illite‐dominated mélange from altered pelagic to hemipelagic sedimentary rocks and chlorite dominated mélange from altered basaltic lithologies. Therefore, to clarify descriptions and interpretation, the two volumetrically dominant matrix types within mélange‐bearing shear zones are referred to as chloritic mélange and illitic mélange for the remainder of this work (Figure 3), after their major phyllosilicate components (Figures 4 and 5).

Chloritic mélange typically comprises variably altered sigmoidal basalt clasts within a strongly foliated chlorite matrix (Figures 4a–4c). Throughout the chloritic mélange, continuous calcite veins up to 1.5 mm thick are oriented ∼15° from the bulk subvertical foliation, in places bisecting basalt clasts (Figures 4a–4c). Calcite veins contain minor quartz and are nearly completely recrystallized to 10–15 μm diameter where they form continuous through‐going volumes (Figures 4d–4f). Calcite away from these veins is coarse‐grained, with curved twins and subgrains of similar size to adjacent recrystallized grains (Figures 4a, 4b, 4d and 4f).

Illitic mélange contains clasts of massive carbonate, variably altered basalt, and fine‐grained sandstone containing quartz, chlorite and albite, within a foliated illite‐rich matrix (Figure 5). The illite matrix contains fine‐grained hematite, giving the illitic mélange a distinctive red appearance and making the matrix opaque in plane‐polarised transmitted light (Figures 5a and 5g). Clasts within illitic mélange typically form layers of elongate, subrounded phacoids within the phyllosilicate foliation (Figures 5a, 5d, 5e and 5f). Throughout the matrix of illitic mélange, multiple generations of variably folded, boudinaged, and fractured chlorite‐quartz‐calcite veins are present at varying angles to foliation (Figures 5a–5g) and have Mg‐rich chlorite along the vein margins (Figures 5f–5h). Opening vectors of veins generally plunge NW, 20–30° from bulk foliation orientation. This means foliation‐parallel veins have a hybrid extensional‐shear opening sense, consistent with the bulk SE‐upward shear sense. Veins at higher angles to foliation have a more extensional opening sense in places bifurcating from foliation‐parallel veins (Figures 5a and 5e). Foliation‐oblique veins are typically dismembered and cut by more continuous foliation‐parallel veins of similar composition (Figures 5a, 5d and 5e). Thin slip surfaces are localized at phacoid margins and locally cut veins (Figures 5a, 5c and 5e). Where some clasts have been boudinaged, veins fill in voids between boudin segments (Figure 5e).

## 6 Formation of the Imbricated Plate Interface

**Figure 6:**
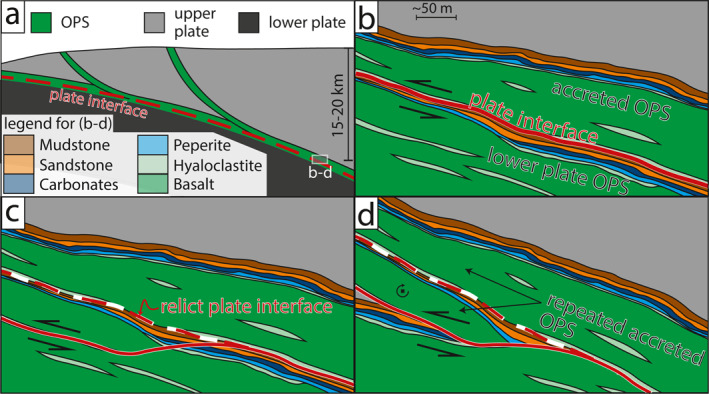
Schematic sketch of the suggested mode of exhumation and rotation of the ocean plate stratigraphy (OPS) ‐ derived tectonic mélange from the plate interface to the outcrop observed at Llanddwyn Island.

Throughout Llanddwyn Island, repeated lenticular slices of OPS containing SE‐younging pillow basalts are bounded by mélange‐bearing shear zones with a SE‐upward shear sense, both consistent with SE‐directed subduction (Figures 1, 2, 3, 4, 5). To imbricate multiple slices, OPS must have been repeatedly delaminated and accreted by sequential downstepping of the plate interface. This likely occurred during subduction‐related deformation by thrusting of submarine volcanics (hyaloclastite and pillow basalt) from the lower part of the OPS over changesiliciclasticpelagic‐hemipelagic sediments from the upper part of the OPS on each mélange shear zone (Figures 1, 6 and 6d; Kimura & Ludden, 1995). Mélange bearing shear zones between lenticular slices are interpreted to represent the plate interface during each episode of downstepping. This repetition of OPS would create a thickened sequence of deep‐ocean volcanic and sedimentary rocks with a volumetrically small component of siliciclasticcs, as seen in the study area.

Chloritic mélange typically bounds the structurally lower NW side of each lens (Figures 1 and 2). In our model, slices of OPS were delaminated along a weak hyaloclastite interval from which most of the chloritic mélange was likely derived. As pillow basalts are sampled within OPS slices at Llanddwyn Island (Figure 1), hyaloclastite must have underlain or intermingled with pillow basalts on the incoming plate prior to subduction. The origin of the matrix chlorite is unclear, though its foliated texture suggests it formed either before or during deformation of the mélange shear zone (Figures 4a, 4c and 4d). It likely formed by interaction of basaltic glass with fluids, either on the sea floor or near the plate interface (Humphris & Thompson, 1978; Kameda et al., 2017; Seyfried & Mottl, 1982).

Most previously studied tectonic mélanges associated with subduction are volumetrically dominated by siliciclastic sequences (Fagereng, 2011b; Fisher & Byrne, 1987; Kimura & Mukai, 1991; Meneghini et al., 2009; Remitti et al., 2011; Rowe et al., 2011; Ujiie, 2002; Vannucchi et al., 2008), suggesting increased sediment thickness at the trench aids in accretion and exhumation. The smaller volumetric component of siliciclastic sediments at Llanddwyn Island shows less sediment was incorporated into the complex during accretion, either due to lower sedimentary input thickness (e.g., at a sediment‐starved margin) or due to preferential incorporation of basaltic material at the plate interface following accretion of overlying turbidites at shallower depth. Repeated delamination of OPS slices along hyaloclastite horizons suggests that the lithostratigraphy of the incoming oceanic volcanic sequence may play a role in dictating the geometry of the plate interface (Figures 1 and 6). That the incoming sequence may control plate interface geometry and slip behavior was recently proposed, also invoking weak volcaniclastic horizons, for the active Hikurangi margin (Barnes et al., 2020). Specifically, recent electrical resistivity surveys across the Hikurangi margin have been interpreted to show thin lenses of altered or volcaniclastic material within the basaltic lower plate (Chesley et al., 2021). Lenses of volcaniclastic material within the basaltic upper oceanic crust may be pre‐subduction analogs of the hyaloclastite layers interpreted here to have been horizons of delamination during subduction (Figures 6c and 6d). It is possible that the exposures on Llanddwyn Island sample a level down‐dip from or deeper than slices of sedimentary material that have not been preserved. However, given that several distinct slices of OPS are juxtaposed on Llanddwyn (Figure 1), with little continentally derived sediment preserved in any of them, we suggest any missing material, if it existed, was accreted rather than involved in plate interface deformation, as is also the case for turbiditic sediments in northern Hikurangi (Barnes et al., 2020).

## 7 Heterogeneous Deformation Within Imbricated Plate Interface Shear Zones

Deformation of mélange shear zones across Llanddwyn Island is heterogeneous because of the variable rheology, composition, thickness (i.e., strain rate) and physical properties (including fluid content and pressure) of each part of the deforming volume. We now characterize the bulk rheology of mélange shear zones and the effects of local heterogeneity on deformation of the plate interface.

### 7.1 Qualitative Bulk Rheology From Grain‐Scale Observations

In both the chloritic and illitic mélanges, the phyllosilicate matrix currently displays the most deformation around and between less deformed clasts of more rigid lithologies (e.g., basalt, sandstone, carbonate; Figures 4 and 5). Phyllosilicates in the mélange matrix are fine‐grained (≤20 μm) and form interconnected anastomosing networks capable of accommodating simple shear deformation by glide or frictional sliding on the basal planes (*sensu* Okamoto et al., 2019). Phyllosilicates have low friction for sliding along their basal planes, so are much easier to deform than clasts (Kronenberg et al., 1990). Clasts within the illitic mélange are commonly sub‐rounded, asymmetrical, and elongate parallel to foliation (Figure 5), consistent with material removal by pressure solution during simple shear (Bos & Spiers, 2001). As material is moved from high to low stress sites along clasts and they elongate parallel to foliation orientation, phyllosilicate foliations become less curved and translation of clasts relative to one another requires lower shear stresses; mélange shear zones therefore weaken with increased strain (Bos & Spiers, 2001). The locally variable clast proportion within both illitic and chloritic mélange likely controls the bulk rheology of mélange shear zones by inhibiting sliding on the matrix where clasts become ‘jammed’, i,e, form a local load‐bearing framework (Beall et al., 2019). Where stress is below that required for brittle shear failure of the clast‐forming material, frictional‐viscous mechanisms combining pressure solution of clasts and frictional or basal slip on phyllosilicates likely dominate the rheology (Bos, 2002; Niemeijer, 2018; Niemeijer & Spiers, 2005). Where phyllosilicate grains are aligned, the rheology of clast‐poor areas likely resembles that of basal sliding on the matrix‐forming phyllosilicate grains (Kronenberg et al., 1990), especially where through‐going shears are present (Handy, 1990). Although mélange deformation localizes in the weak matrix, it is known from engineering studies (Medley, 2001) and numerical models (Beall et al., 2019) that increasing clast proportion increases bulk strength by enforcing shear surface curvature and increasing local stress and strain rate.

### 7.2 The Role of Veins: Strain Hardening and Variable P_*f*_

Veins up to several mm thick are common within both illitic and chloritic mélange (Figures 4 and 5). Formation of these veins likely accompanied foliation‐parallel extension (Schmalholz & Maeder, 2012), with quartz‐calcite precipitation cementing fractures initiated by extension‐fracture boudinage. These veins therefore strain‐hardened a through‐going horizon before gradually weakening where the vein was disaggregated.

**Figure 7:**
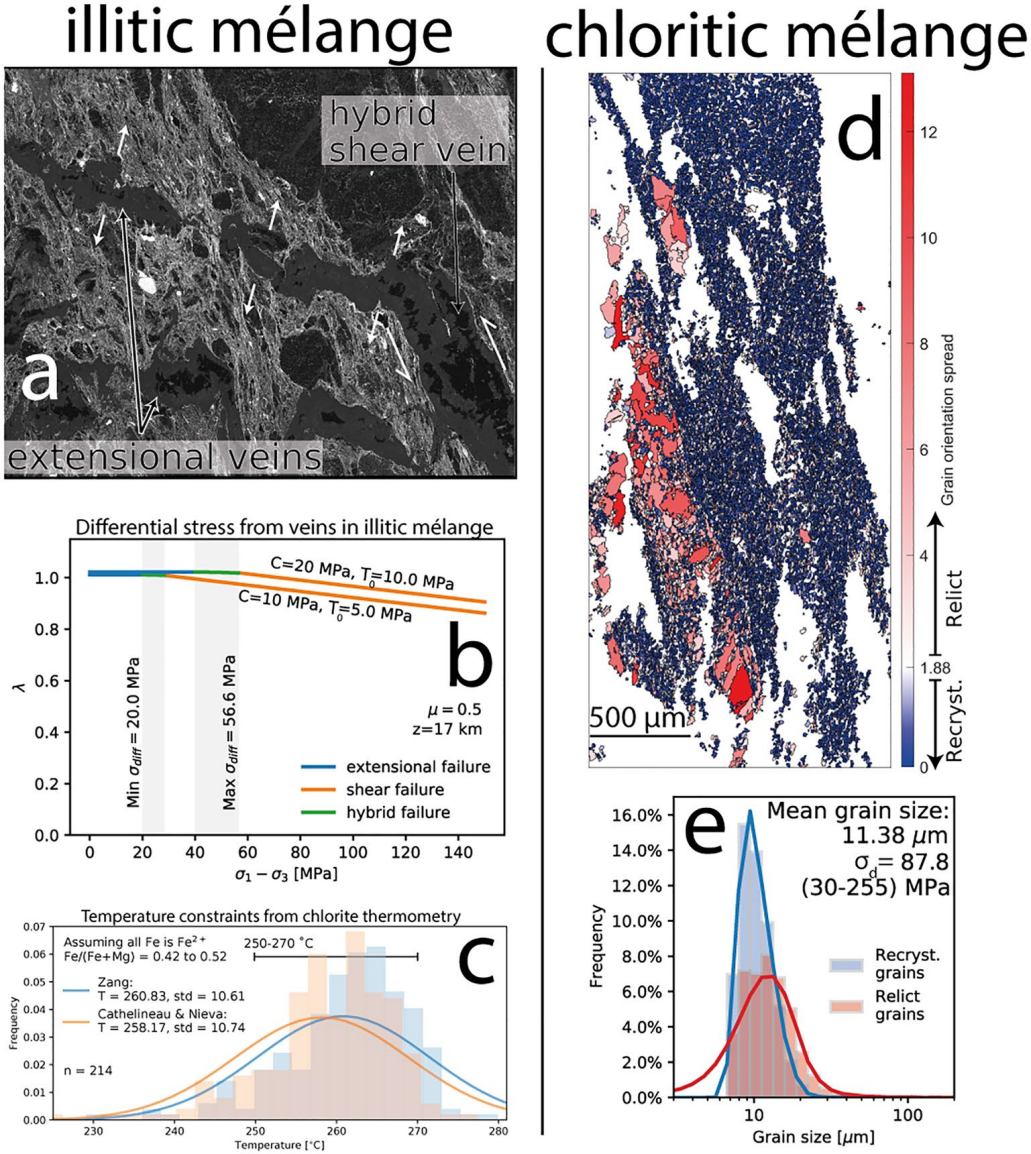
Differential stress‐temperature constraints from both mélanges. (a) BSE image of hybrid extensional‐shear veins in the illitic mélange where white arrows are opening vectors, (b) differential stress‐pore fluid factor estimates from analysis of hybrid extensional vein failure of the illitic mélange (*μ* = 0.5, *z* = 17 km), (c) histogram and fitted probability distribution function of temperatures from the chlorite geothermometers of Cathelineau and Nieva (1985) and Zang and Fyfe (1995) applied to chlorite in syn‐kinematic hybrid extensional‐shear veins, (d) EBSD reconstructed grain map colored by grain orientation spread (GOS) in degrees. Threshold GOS value was calculated following the method of Cross et al. (2017), (e) grain size histogram and fitted probability distribution function for relict (red) and recrystallized (blue) grains.

Opening vectors of the foliation‐parallel veins in the illitic mélange show they accommodated opening and shear (Figures 4a and 5a), requiring pore fluid factors (*λ*) of 1–1.05 at the instant of formation (Figures 7a and 7b; Cox, 2011). *λ* is calculated as *λ* = *P*_*f*_/*σ*_*v*_, meaning at hydrostatic *P*_*f*_, *λ* ≈ 0.4, and at lithostatic *P*_*f*_, *λ* = 1. The source of the solute is not clear, but local pressure solution occurring on clasts or longer‐range fluid flow within the mélange are both candidates. Several generations of variably deformed veins are present within the illitic mélange, suggesting pore fluid pressures were cyclically elevated to supra‐lithostatic levels during deformation (Figures 5a and 5d). Because the vein fill is stronger than the mélange matrix, the illitic mélange episodically strengthened with each pore fluid pressure cycle as vein material precipitated (Figure 7b). Quartz and calcite have opposite solubility relationships with temperature (Plummer & Busenberg, 1982; Rimstidt & Barnes, 1980), meaning it is unlikely that precipitation in the veins was driven by a temperature change. It is more likely that precipitation occurred due to either long range pressure solution or pressure drops (*cf.* Meneghini & Moore, 2007). Mineral precipitation due to pressure drop is consistent with short‐lived fluid pressure pulses forming veins by hydrofractures that rapidly fill with quartz and calcite as pore fluid pressure reverts toward hydrostatic (Fisher & Brantley, 2014; Ujiie et al., 2018). Whether veins partially or totally fill during each hydrofracture event is unclear, but the lack of crack‐seal textures suggests veins were not systematically re‐fractured. The lack of evidence for re‐fracturing is consistent with veins representing strain‐hardening horizons and causing slip localisation elsewhere, as also suggested for other vein networks in relatively weak host rocks (Fagereng, 2011a; Holland & Urai, 2010). Magnesium‐rich marginal chlorite within the veins could have formed by either precipitation or reaction with the mélange matrix (Figures 5f–5h), though the lack of magnesium‐rich species within the matrix suggests the former.

Chloritic mélange also hosts quartz‐calcite veins, though calcite volumetrically dominates these (Figures 4 and 7d). Unlike veins in the illitic mélange, veins in the chloritic mélange are not boudinaged or disaggregated, suggesting they deformed at similar strain rates to the mélange matrix (Figure 4a). Calcite within the veins is almost completely recrystallized to ∼12 μm (Figures 4, 5, 6, 7 and 7e), which may have facilitated deformation by a combination of grain‐size sensitive (*sensu* Herwegh et al., 2003) and dislocation creep (Renner et al., 2002). Whereas grain size sensitive creep of recrystallized calcite is promoted by secondary phases such as quartz (Herwegh et al., 2003), dislocation creep and grain growth of calcite in these veins would have been inhibited (i.e., required greater stresses to achieve the same strain) by greater amounts of quartz pinning calcite grain boundaries, similar to calcite within veins throughout the illitic mélange. Composition of the vein filling, resulting from fluid chemistry at the instant of fracture formation, therefore exhibits a demonstrable effect on the bulk strength of the mélange where veining is common. This is exemplified by the deformation of calcite‐rich veins in the chloritic mélange to high strains and the disaggregation of less deformed quartz‐rich veins in the illitic mélange (Figures 4 and 5).

## 8 Quantifying Rheology From Observations

Observations of chloritic and illitic mélange units show that both experienced distinct modes of shear deformation. Within the chloritic mélange, calcite veins were deformed to shear strains of ≥4 and the chlorite matrix appears deformed to moderately high shear strains (Figure 4). Shear deformation in the illitic mélange appears to have dominantly occurred by slip on illite in the matrix, aided by foliation‐normal pressure solution of clasts allowing translation of clasts, and was cyclically punctuated by transient hybrid extensional‐shear veining. We now use several methods to calculate the differential stress and temperature during deformation, and model the relative contributions of matrix and clast deformation within each mélange unit to the overall strain rate and slip velocity of the shear zone.

### 8.1 Differential Stress and Temperature Estimates on Gwna Complex Deformation

Different methods were used to estimate differential stresses (*σ*_*d*_) and temperature (*T*) for illitic and chloritic mélanges. Calcite veins throughout the chloritic mélange were analyzed with EBSD and recrystallized grains were distinguished from relict grains based on their spread of orientations relative to a threshold value of 1.88° (Cross et al., 2017). We used a nominally monomineralic area of the calcite veins to minimize the effects of grain pinning by secondary phases. The mean size of recrystallized calcite grains was then used to determine *σ*_*d*_ during steady‐state vein deformation using the paleopiezometer of Platt and De Bresser (2017). Recrystallized grains have a mean grain size of 11.4 μm, corresponding to a *σ*_*d*_ of 88 MPa. Ranges in exponent values in the method of Platt and De Bresser (2017) cause broad 95% confidence limits, between 30 and 255 MPa (Figure 7e). Assuming synkinematic recrystallization of these veins, this measurement reflects the background *σ*_*d*_ during recrystallization of calcite. From optical microscopy it appears other calcite veins throughout the chloritic mélange have similar recrystallized grain size.

Foliation‐parallel calcite‐quartz‐chlorite veins accommodated both extensional and shear offset (Figures 5d and 5e). Foliation‐oblique veins are continuous with those oriented parallel to foliation with the same opening sense (Figure 7a), suggesting that they formed simultaneously, and differences in orientation reflect local variations in cohesion and tensile strength. Assuming typical values for cohesion (*C*) and tensile strength (*T*_0_) from accretionary complex sediments (*C* = 10 − 20 MPa, *T*_0_ = 5 − 10 MPa; Schumann et al., 2014), a *λ* versus *σ*_*d*_ plot (*sensu.* Cox, 2011) shows the *σ*_*d*_ range over which these hybrid extensional shear veins might have occurred (Figure 7b). Hybrid extensional‐shear veins should occur at supra‐lithostatic pore fluid pressures and *σ*_*d*_ in the range between 4*T*_0_ and 5.66*T*_0_ (Secor, 1965), the assumed *C* and *T*_0_ strength values therefore constrain the *σ*_*d*_ range at the instant of hybrid vein formation to between 20 and 57 MPa (Figure 7b). As we do not know the actual values for *C* or *T*_0_, we take this range (38 ± 18 MPa) as the estimated *σ*_*d*_ at the instant of hybrid fracture formation.

Whereas the timing of chlorite formation in the chloritic mélange matrix is unclear, chlorite in many variably deformed veins throughout the illitic mélange (Figures 5f–5h) must have crystallized during vein formation, allowing us to use geothermometry to discern temperatures during deformation having interpreted the veins as synkinematic that is, related to syn‐subduction juxtaposition of underplated ocean floor sequences. We quantified the composition of 214 vein‐hosted areas (50 − 100 μm^2^) of chlorite from the illitic mélange using SEM‐EDS and calculated temperatures of crystallisation using the geothermometers of Cathelineau and Nieva (1985) and Zang and Fyfe (1995) as they were calibrated over similar *Fe*/*Mg* ratios (0.42–0.52). This analysis yielded two temperature distributions with means of 261 and 258°C for the geothermometers of Zang and Fyfe (1995) and Cathelineau and Nieva (1985), respectively, and standard deviations of 11°C for both (Figure 7c). These temperatures are consistent with both (a) subgreenschist metamorphism and the preservation of original lithological texture visible across the island and (b) the onset of recrystallization and crystal‐plastic deformation of calcite (Bauer et al., 2018; Kennedy & White, 2001). We therefore take ∼260 ± 10°C as the approximate temperature of deformation.

Average differential stress estimates from recrystallized calcite grain size and the hybrid failure criterion yield very different values (∼88 and 38 MPa, respectively; Figure 7). Uncertainty ranges for the two values (30–255 and 10–57 MPa, respectively) overlap between 30 and 57 MPa due to poorly constrained exponents in the recrystallized calcite piezometer of Platt and De Bresser (2017). Errors in the cohesion and tensile strength values are unknown (Schumann et al., 2014). Differences between stress estimates may be expected as recrystallized calcite grain size is used to estimate a steady state *σ*_*d*_ during recrystallization and the hybrid failure criterion estimates *σ*_*d*_ at the instant of failure. Previously, variable *σ*_*d*_ estimates within a shear zone have been interpreted to represent the stress within that phase (Stenvall et al., 2019). Similarly, it may be that recrystallization in a through‐going calcite vein that is deforming slowly, and provides a barrier to matrix shear records a higher differential stress than required for episodic, fluid‐driven brittle failure within the more rapidly deforming matrix (Beall et al., 2019). As the *σ*_*d*_ estimate from recrystallized calcite grain size with the piezometer of Platt and De Bresser (2017) likely represents stresses during uniform background creep, it is therefore used to interpolate velocities in the following modeling.

### 8.2 Modeling Stress and Strain Rate in Chloritic and Illitic Mélange

Given the evidence for various deformation mechanisms operating under variable pore fluid pressure (Section 7.2), we now quantify the relative contributions of clast and matrix deformation using shear stress (*τ*)‐strain rate $\left(\dot{\gamma }\right)$ (then velocity *V*) curves following the approach outlined by French and Condit (2019). This assumes an idealized plate interface shear zone (Figure 3a) dipping at 10° (*δ*) and deforming at *T* = 260°C (based on chlorite geothermometry; Figure 7c). We assume an interface depth of 17 km, consistent with the estimate of *T* from chlorite geothermometry on a 15°C km^−1^ subduction zone geotherm, and a density of the upper crust of 2,750 kg m^−3^ to calculate a vertical stress (*σ*_*v*_) of 458 MPa. The maximum principal stress (*σ*_1_) was assumed to plunge at an angle (*ψ*) of 35°, antithetic to the plate interface, the minimum principal stress (*σ*_3_) plunges 90° from this, synthetic to the plate interface, making the interface a plane of maximum shear stress (See Figure 2 in French & Condit, 2019, for summary).

We followed both Bletery et al. (2017) and French and Condit (2019) in modeling the *τ* required for frictional slip at a given *V* as

(1) $$\tau =\frac{\mu \left(T,V\right)\left(\sigma_{v}-P_{f}\right)\sin\left(2\delta +2\psi \right)}{\sin\left(2\delta +2\psi \right)+\mu \left(T,V\right)\left(\cos\left(2\delta +2\psi \right)-\cos\left(2\psi \right)\right)}$$

where *μ*(*T*, *V*) is the friction coefficient of the material at temperature *T* and velocity *V*. This was calculated using

(2) $$\mu \left(T,V\right)=\mu_{0}+{\left({d\mu }_{ss}/dlnV\right)}_{T}\ln\frac{V}{V_{0}}$$

where *μ*_0_ is the friction coefficient at temperature *T* and pre‐step slip velocity *V*_0_. The rate‐and‐state parameter $\left({\left({d\mu }_{ss}/dlnV\right)}_{T}\right)$ determines the response of *μ*(*T*, *V*) with a change from *V*_0_ to *V* at temperature *T*(Chester, 1994); if ${\left({d\mu }_{ss}/dlnV\right)}_{T}$ is less than 0 then *μ*(*T*, *V*) reduces with increased velocity and the material velocity‐weakens, if ${\left({d\mu }_{ss}/dlnV\right)}_{T}$ is greater than 0 then *μ*(*T*, *V*) increases with increased velocity and the material velocity‐strengthens. Friction was modeled at constant *T* and variable *V*, meaning values for *μ*(*T*, *V*) were controlled by initial inputs of *V*_0_, *μ*_0_, and ${\left({d\mu }_{ss}/dlnV\right)}_{T}$. To retain comparability from experimental data, values for *μ*_0_ and ${\left({d\mu }_{ss}/dlnV\right)}_{T}$ were selected if they were measured at velocity steps originating with a *V*_0_ of 1 μm s^−1^. The experimental data we use did not measure *μ*_0_ and ${\left({d\mu }_{ss}/dlnV\right)}_{T}$ for calcite and chlorite at the modeling *T* so, following den Hartog et al. (2012), values were linearly interpolated between data measured at temperatures above and below 260°C.

**Figure 8:**
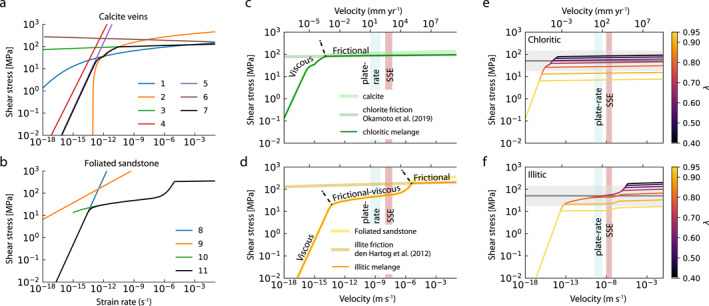
Modeled shear stress‐shear strain rate (and velocity) curves for chloritic and illitic mélange. Modeled curves for (a) calcite are 1‐dislocation creep from Renner et al. (2002), 2‐dislocation creep from Rutter (1974), 3‐grain size‐insensitive cross slip from De Bresser (2002) in the exponential form fitted by Verberne et al. (2015), 4‐diffusion creep from Herwegh et al. (2003), 5‐pressure solution from Bos (2002), 6‐frictional slip from Verberne et al. (2015), 7‐combined *τ*‐$\dot{\gamma }$ curve for calcite; (b) quartz are 8‐pressure solution from (Rutter, 1976), 9‐dislocation creep from (Lu & Jiang, 2019), 10‐frictional‐viscous flow of a quartz‐phyllosilicate aggregate (Bos, 2002; Niemeijer & Spiers, 2005), 11‐combined *τ*‐$\dot{\gamma }$ curve for quartz. Panels c and d show curves for combined calcite with chlorite (representing chloritic mélange) and combined quartz with illite (representing illitic mélange), respectively. Chlorite and illite strengths were modeled using frictional parameters from Okamoto et al. (2019) and den Hartog et al. (2012), respectively. Overall curves for parts c and d represent the mechanism of minimum shear stress at hydrostatic pore fluid factor (*λ* = 0.4) for chloritic and illitic mélange, respectively. Plots e and f show these overall curves for each melange at variable *λ*. Red and blue shaded areas correspond to velocity ranges for plate convergence (10–100 mm yr^−1^) and slow slip (SSE) (300–1,500 mm yr^−1^), respectively. Modeling follows the methodology of French and Condit (2019) on an interface dipping 10° at 260°C and 17 km depth.

For the chloritic mélange, *τ*‐$\dot{\gamma }$ curves for veins were modeled for pressure solution (Bos, 2002), diffusion creep (Herwegh et al., 2003), dislocation creep (Renner et al., 2002; Rutter, 1974), grain size insensitive cross slip (De Bresser, 2002), and frictional sliding of calcite (Verberne et al., 2015) (Figure 8a). Following French and Condit (2019), dislocation creep of calcite was modeled using the flow law of Renner et al. (2002) at lower values of *τ* and the flow law of Rutter (1974) at higher values of *τ*, this transition occurs at ∼35 MPa at 260°C. Verberne et al. (2015) measured calcite friction values at velocity steps of 1–3 μm s^−1^, temperatures of 20–600°C, and effective normal stresses ($\sigma_{\mathrm{eff}}^{n}$) of 30, 50, 80, and 100 MPa. At *λ* = 0.8, $\sigma_{\mathrm{eff}}^{n}$ on the modeled plate interface were ∼97 MPa, making a $\sigma_{\mathrm{eff}}^{n}$ value of 100 MPa the most appropriate here. Values of *μ*_0_ and ${\left({d\mu }_{ss}/dlnV\right)}_{T}$ were linearly interpolated at 260°C to yield values of 0.49 for *μ*_0_ and ‐0.00637 for ${\left({d\mu }_{ss}/dlnV\right)}_{T}$. This suggests calcite at this temperature would be relatively strongly velocity‐weakening and have a high initial strength relative to the surrounding chlorite. For the chlorite matrix we assumed frictional sliding to be the dominant mechanism. Okamoto et al. (2019) measured friction values at velocity steps of 1–3 μm s^−1^. Values at *λ* = 0.4 were interpolated from measurements at 200 and 300°C to give a *μ*_0_ of 0.28 and a ${\left({d\mu }_{ss}/dlnV\right)}_{T}$ of 0.001258 at 260°C. This suggests chlorite at this temperature would be velocity‐strengthening, and have a lower initial strength than calcite. Individual *τ*‐$\dot{\gamma }$ or *V* curves for mechanisms modeled in the chloritic melange are shown in Figures 8a and 8c.

The illitic mélange has clasts of varied composition including basalt, carbonate, and foliated sandstone containing quartz, albite, and chlorite (Figures 5f and 5h). Quartz deformation was modeled using pressure solution creep (Rutter, 1976) and dislocation creep (Lu & Jiang, 2019), combined with a flow law for the frictional‐viscous flow of a quartz‐phyllosilicate aggregate (Bos, 2002; Niemeijer & Spiers, 2005). Albite‐rich phacoids in the illitic mélange are elongate parallel to foliation (Figure 5), consistent with minor flattening by pressure solution (Bos, 2002). Due to the lack of available data for albite solubility and its low‐T deformation mechanisms, we assumed a quartz‐dominated sandstone clast lithology at hydrothermal conditions approximating the 260°C temperature and fluid‐present conditions of deformation in the study area. Quartz is more easily deformed than albite under hydrothermal conditions (He et al., 2013); by excluding deformation of albite‐rich phacoids our models likely provide a lower bound on the real strength of the illitic mélange. The frictional behavior of the illite matrix surrounding the clasts was modeled using values of *μ*_0_ = 0.52 and ${\left({d\mu }_{ss}/dlnV\right)}_{T}=0.00484$ from den Hartog et al. (2012), measured at a velocity step of 1–10 μm s^−1^ and a *T* of 250°C, within error from the chlorite geothermometry. This suggests illite at this temperature would be both velocity‐strengthening and relatively strong for a phyllosilicate. Individual *τ*‐$\dot{\gamma }$ or *V* curves for mechanisms modeled in the illitic melange are shown in Figures 8b and 8d.

**Figure 9:**
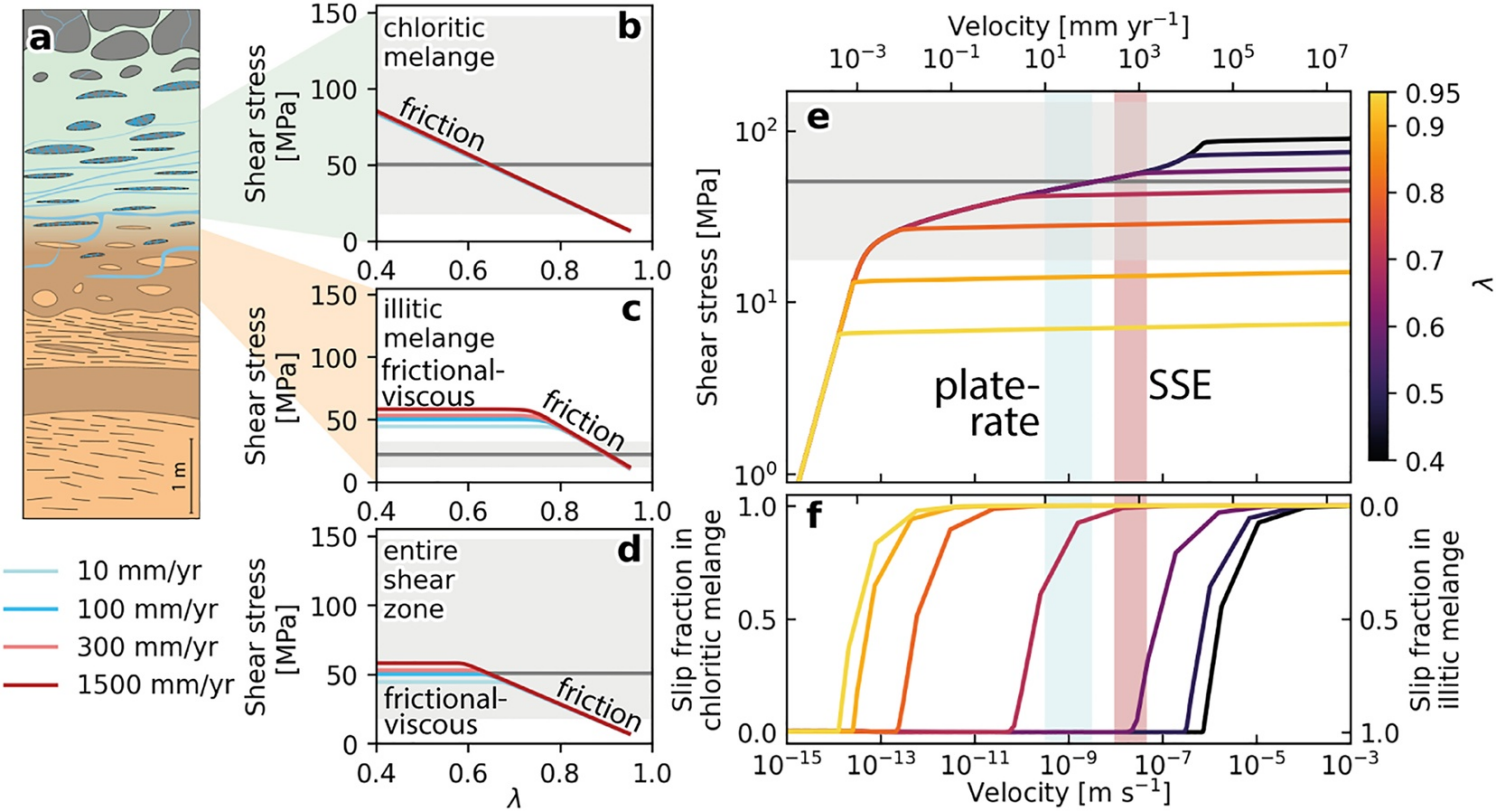
Modeled effects of velocity (*V*) and pore fluid factor (*λ*) on deformation shear stress (*τ*). (a) Idealized shear zone used for model geometry, (b)–(d) *τ*‐*λ* curves for chloritic mélange (b), illitic mélange (c), and the entire shear zone (d) at velocities corresponding to lower and upper limits of plate convergence (10–100 mm yr^−1^, blue fill in (e) & (f) and slow slip (300–1,500 mm yr^−1^, red fill in (e) & (f) velocities (Bürgmann, 2018), (e) *τ*‐*V* curves at various *λ* values for the entire shear zone, (f) slip fraction in each mélange versus total shear zone velocity at various *λ* values. Gray shaded areas and lines are converted differential stresses ranges and estimates in either the chloritic (b), illitic (c), or combined (d) & (e) mélanges, see Figure 7 and Section 8.1 for details. Gray horizontal lines and fill show average and range of stresses from calcite piezometry (b, d, & (e) or hybrid extensional failure criterion (c). *τ*‐$\dot{\gamma }$ or *τ*‐*V* curves for individual model components are shown in Figure 8.

Curves for *τ*‐$\dot{\gamma }$ from different mechanisms were combined by assuming additive shear strain rate deformation at a given shear stress, and vary for each mélange with varying *λ* (Figures 8e and 8f). We have, throughout, assumed that deformation occurs by the mechanism requiring the smallest *τ*. Where appropriate, *V* was converted to $\dot{\gamma }$ by dividing by the unit thickness in our idealized shear zone (Figure 9a) and converting $\dot{\gamma }$ to *V* was achieved by performing the inverse. Thicknesses used in the modeling are (Figure 9a): calcite veins = 1 mm, chlorite matrix = 2 m, illite matrix = 1.2 m, sandstone clasts = 1 m.

### 8.3 Shear Stress, Strain Rate, and Velocity of Modeled Chloritic and Illitic Mélange at Variable Pore Fluid Pressure

Cross‐cutting and variably deformed hybrid extensional‐shear veins indicate that elevated pore fluid pressures (*P*_*f*_) were cyclically reached during shear deformation within the illitic mélange (Figures 5 and 7). Increases in *P*_*f*_ reduce $\sigma_{\mathrm{eff}}^{n}$ and consequently *τ* required to activate deformation mechanisms sensitive to $\sigma_{\mathrm{eff}}^{n}$, such as frictional sliding. Pressure solution is sensitive to the stress gradients around grains, controlled by $\sigma_{\mathrm{eff}}^{n}$ and *τ*, so is also sensitive to $\sigma_{\mathrm{eff}}^{n}$ (Bos, 2002). Grain size sensitive and insensitive creep, which occur at low strain rates and velocities, are not sensitive to $\sigma_{\mathrm{eff}}^{n}$. The *τ* required for viscous creep at a given velocity is therefore unaffected by variations in *P*_*f*_. On the other hand, the *τ* required for viscous creep depends critically on the velocity. The stress‐dependence of viscous creep with changes in velocity is controlled by the stress exponent used in the flow law. Stress exponents for creep mechanisms are generally low, meaning stresses required for deformation at a given strain rate is highly sensitive to the rate of deformation (1.1 for grain size sensitive creep and 2 for grain size insensitive creep of calcite; Herwegh et al., 2003; Renner et al., 2002). For comparison, consistent frictional sliding on slightly velocity‐strengthening materials has a *τ*‐*V* relationship consistent with a stress exponent of >30, meaning large changes in velocity occur with relatively small changes in applied stress.

In the modeled chloritic mélange, frictional sliding on the matrix chlorite requires lower shear stresses than viscous deformation of calcite at *V* ≥ 1 μm yr^−1^ and dominates with increased velocity well into seismic slip rates of 1 m s^−1^ (Figure 8c), though we do not account for seismic slip weakening (Di Toro et al., 2011). At aseismic creep velocities consistent with plate convergence (10–100 mm yr^−1^) and geodetically modeled slow slip events (300–1,500 mm yr^−1^; Bürgmann, 2018), modeled chloritic mélange deformation occurs by slightly velocity‐strengthening frictional sliding and is highly sensitive to pore fluid pressure (Okamoto et al., 2019). Shear stresses at velocities of 10–1,500 mm yr^−1^ in the chloritic mélange decrease linearly from 83 to 85 MPa at *λ* = 0.4 to 6–7 MPa at *λ* = 0.95, with very little contribution from viscous or frictional deformation of calcite (Figure 9b).

In the illitic mélange, slip at plate rate and slow slip velocities occurs by frictional‐viscous deformation of sandstone clasts and frictional sliding on illite with the governing mechanism dictated by the pore fluid pressure. Frictional‐viscous deformation of sandstone clasts is less sensitive to *P*_*f*_ changes than frictional sliding (Figure 9c). Modeled frictional‐viscous deformation of sandstone clasts, which dominates at *λ* < 0.75, is strongly velocity‐strengthening (Bos, 2002; Niemeijer, 2018; Niemeijer & Spiers, 2005). Shear stresses at velocities of 10–1,500 mm yr^−1^ in the illitic mélange are therefore 44–58 MPa at 0.4 ≤ *λ* < 0.75 (Figure 9c). At the same velocities and *λ* ≥ 0.75, frictional sliding of the illite matrix requires less shear stresses than frictional‐viscous deformation and modeled shear stresses reduce linearly from 44‐58 MPa to 10–12 MPa (Figure 9c).

When considering the shear zones as a whole, deformation is assumed to localize into the component where displacement requires the lowest shear stress. At plate convergence and slow slip velocities, strongly rate‐strengthening frictional‐viscous deformation in sandstone clasts of the illitic mélange dominates at shear stresses of 44–58 Mpa and *λ* < 0.65 (Figures 9c and 9d). At *λ* ≈ 0.65, slightly velocity‐strengthening frictional sliding on chlorite in the chloritic mélange occurs at shear stresses of ∼50 MPa, decreasing to 10–12 MPa at *λ* = 0.95 (Figures 9b and 9d). At velocities below ∼1 μm yr ^−1^ and *λ* < 0.9 slip occurs by strongly rate‐strengthening viscous deformation of sandstone clasts in the illitic mélange (Figure 9e). As shear stresses required for frictional sliding of chlorite decrease the most with increasing P_*f*_, it becomes the dominant mechanism with increasing *λ* (Figure 9e). Slip partitioning between mélange units therefore depends on both velocity and *λ*; slip at low *V* and *λ* occurs dominantly within the illitic mélange and slip at higher *V* and *λ* occurs dominantly in the chloritic mélange (Figure 9f). Between these conditions, slip occurs in both mélanges over a continuous transition at a range of *V* controlled by *λ*. In the idealized shear zone modeled here, deformation at plate convergence and slow slip velocities occur by (a) frictional sliding on chlorite in the chloritic mélange at 1 > *λ* > 0.7, (b) frictional‐viscous deformation of sandstone clasts in the illitic mélange at *λ* < 0.6, or (c) a combination of both mechanisms in both mélanges at 0.6 < *λ* < 0.7 (Figure 9f).

### 8.4 Comparing Differential Stress and Pore Fluid Factor Estimates With Modeled Velocities

Constraints from recrystallized calcite within veins in the mélange shear zones yielded differential stresses for viscous creep of 88 MPa with an uncertainty range of 30–255 MPa. Using $\tau =\sigma_{d}/\sqrt{3}$ (Nye, 1953), this corresponds to a shear stress of 51 MPa with an uncertainty range of 17–147 MPa. With this shear stress and hydrostatic pore fluid pressures (*λ* = 0.4), frictional‐viscous slip in foliated sandstone clasts within the illitic mélange occurs at *V* < 1 × 10^−9^ m s^−1^ (Figures 9d and 9e). At the same shear stresses and *λ*, calcite in the chloritic mélange is modeled to deform by dislocation creep at low strain rates ($\dot{\gamma }\approx 1\times 10^{-12}$ s^−1^; Figure 8a) and shear stresses are not high enough to cause frictional slip in the chlorite matrix. This is consistent with observations, as widespread calcite recrystallization (Figure 4) requires dislocation creep to be active to form subgrain walls (Cross & Skemer, 2019; Poirier & Guillopé, 1979; White, 1977). At shear stresses estimated from the hybrid failure criterion in the illite matrix (∼22 MPa) the illitic mélange is modeled to deform by pressure solution at rates below plate‐rate (Figures 8b and 8d), consistent with observed flattening of clasts within the illitic mélange or brittle deformation at high *λ*. At near‐lithostatic pore fluid pressures (*λ* = 0.9), frictional slip at shear stresses of 51 MPa in chlorite occurs at modeled slip rates exceeding 1 m s^−1^ in the chloritic mélange (Figures 9e and 9f). Slip in the illitic mélange under the same conditions occurs by frictional sliding at rates intermediate between slow slip and seismic slip (Figures 9e and 9f).

For stress constraints from the subduction complex and velocity constraints from modern margins to agree, sub‐lithostatic pore fluid factors (between 0.6 and 0.7; Figure 9e) or strain‐rate dependent deforming thicknesses are required. Elevated pore fluid factors are evidenced by abundant veining throughout deformed zones (Figures 3, 4, 5), suggesting this occurred episodically in the mélanges now exposed at Llanddwyn Island. The veins may be related to slow deformation but localized slip surfaces adjacent to clasts throughout the illitic mélange suggest localized, relatively rapid (above plate‐rate) slip occurred, likely by episodic weakening at elevated *P*_*f*_ (Figure 5). Differential weakening at pore fluid factors between hydrostatic and lithostatic suggests slip on the plate interface will variably partition with increased *P*_*f*_ and frictional or compositional heterogeneity.

### 8.5 Slip Instabilities During Frictional Sliding at Elevated *P*_*f*_

For slip to transiently accelerate from plate‐rate to slow slip velocities, it has been widely suggested that transient instabilities during slip must alter the rheology of the plate interface (Bürgmann, 2018). These instabilities are as yet poorly understood, but are likely dominantly controlled by the deforming geometry and composition within the plate interface shear zone leading to conditionally dependent values of the rate‐and‐state parameter (${\left({d\mu }_{ss}/dlnV\right)}_{T}$; Equation 2). One such example of a conditionally sensitive instability is *P*_*f*_‐dependent variability in the critical nucleation length recorded at depths near the up‐dip limit of the seismogenic zone by Phillips et al. (2020). This variability would mean that as *P*_*f*_ increases, the slow slip becomes more likely than unstable seismic slip in chlorite bordering basalt clasts. This increased propensity for slow slip requires mixing of velocity‐strengthening illite and velocity‐weakening altered basalt at the appropriate conditions (Phillips et al., 2020). At the estimated temperature of 260 ± 10°C both chlorite and illite are velocity‐strengthening (Den Hartog & Spiers, 2014; Okamoto et al., 2019) but mixtures of phyllosilicates and soluble phases (e.g., quartz, calcite) can be velocity‐weakening at certain temperatures and velocities (Niemeijer, 2018). Accordingly, some chlorite‐bearing gouges with major components of quartz and feldspars have been determined as velocity‐weakening at *T* > 200°C (Boulton et al., 2014), but no through‐going discrete slip surfaces are recognised within the chloritic mélange described here. At a larger scale, the adjacent shearing of chloritic and illitic mélange along shear zones at the base of delaminated oceanic crust may allow for mixing of velocity‐strengthening and velocity‐weakening materials, suggesting greater involvement of altered oceanic material in plate interface deformation could increase the propensity for slow slip.

Foliation‐parallel veins within the illitic mélange are consistent with cyclical formation at transiently elevated *P*_*f*_ (Figures 5, 6, 7). Modeling suggests that between episodic vein formation, at estimated differential stresses of ∼88 MPa and *P*_*f*_ < 0.75, the illitic mélange was creeping by frictional‐viscous pressure solution‐mediated creep (Figure 9c), which is also illustrated by the microstructures of the illitic mélange (Figure 5). Slow viscous creep of phyllosilicates between clasts would reduce pore volume there, locally increasing *P*_*f*_ (van den Ende et al., 2020; Ikari et al., 2009). Locally increased *P*_*f*_ would reduce frictional resistance, increasing the likelihood of granular flow of clasts within the illitic mélange (Bos et al., 2000). Following van den Ende et al. (2020), when and where shear stresses are high enough to cause granular flow it is accompanied by increased porosity between clasts, rapidly reducing shear strength and weakening in the illitic mélange. If the melange has a sufficiently low stiffness, a frictional instability would be generated and fault slip would accelerate, transiently increasing porosity along the sliding surface (van den Ende et al., 2020). This porosity increase would be associated with fluid propagation along the slip surface, and the resultant pressure drop would lead to precipitation of quartz‐calcite‐chlorite veins (e.g., Figures 5f–5h). Precipitation of the vein along the slip surface would harden it and reduce porosity, arresting slip. This could be repeated numerous times with slow creep deforming veins between slip events to produce the variably deformed veins throughout the illitic mélange (Figures 5f–5h). Hybrid extensional‐shear veins observed within the illitic mélange are consistent with this hypothesis, suggesting viscous creep drives instabilities through porosity variations that affect P_*f*_. Viscous creep is more common on the deeper plate interface at higher temperatures, suggesting this mechanism may also occur there, essentially as proposed by Menegon et al. (2015). Elevated P_*f*_ could also be caused by local fluid production or influx, but separating these drivers is difficult. As shown by the combined field and modeling approach taken here, the generation of instabilities within a heterogeneous creeping shear zone could occur by various material and texture‐dependent mechanisms, and discerning how these processes operate within natural shear zones to generate slip transients is an exciting new challenge.

## 9 Conclusions

The plate interface shear zones represented at Llanddwyn Island are locally variable and heterogeneous, having formed <15 m thick shear zones which were then imbricated over tens to hundreds of meters. Imbricated shear zones host tectonic mélange units derived from the deformation of both siliciclastic sediments and altered volcaniclastics. Illitic mélange, likely derived from siliciclastic material, has a block‐in‐matrix texture with clasts of varied composition cut by variably deformed cross‐cutting quartz‐chlorite‐calcite veins. Geothermometry on synkinematic chlorite within veins in the illitic mélange yields a temperature of 260 ± 10°C. Chloritic mélange, likely derived from altered volcanic material, has a chlorite matrix and altered basalt clasts, with recrystallized through‐going calcite veins deformed to shear strains of 4–5. Both mélange units have deformed by slip on phyllosilicates in the matrix, but the rheology of each have been altered by syn‐kinematic veining. Transient supra‐lithostatic pore fluid factors must have occurred cyclically during deformation of the illitic mélange to form multiple generations of strain hardening, variably boudinaged veins.

Shear stress‐strain rate/velocity curves were modeled for each component of the illitic and chloritic mélanges using published flow laws for viscous mechanisms and rate‐and‐state friction parameters measured under in‐situ conditions (Figure 8). At hydrostatic pore fluid pressures (*λ* = 0.4), viscous deformation mechanisms (crystal plasticity, pressure solution) dominate and modeled slip rates only agree with those required for plate boundary deformation in meter‐thick siliciclastic quartz under the highest differential stress estimate from the shear zone. At near‐lithostatic pore fluid pressures (*λ* = 0.95), frictional slip is distributed throughout matrix‐forming chlorite and vein‐bound calcite and associated with transient slip velocity increases (Figures 9e and 9f). To explain observed deformation microstructures, time‐variable but sub‐lithostatic pore fluid pressures are required and deformation occurred at velocities fluctuating between slower than average plate deformation rate and episodically elevated slip speeds. Modeling shows heterogeneous materials within a plate interface shear zone likely undergo differential weakening at elevated *P*_*f*_, suggesting that slip partitioning varies in response to changes in fluid pressure.

Slip transients such as slow slip events require instabilities to develop during otherwise steady, slow creep. Compositional or textural constraints observed within shear zones on Llanddwyn Island indicate these instabilities may develop by local *P*_*f*_ variations induced by pore compaction during creep or in response to influx and episodic release of fluids, or a combination of these processes. Multiple generations of cross‐cutting foliation‐parallel quartz‐calcite‐chlorite veins within the illitic mélange are consistent with pressure drops caused by repeated instability‐driven slip transients at elevated *P*_*f*_.

## Data Availability

Tabulated chlorite EDS data are available at doi:https://doi.org/10.5281/zenodo.6960141. Reviews by J. Wakabayashi, an anonymous reviewer, and Associate Editor P.G. DeCelles significantly improved the manuscript.
